# Supplementary material for: Rapid turnover of life-cycle-related genes in the brown algae
Source: Genome Biol. 2019 Feb 14;20:35. doi: 10.1186/s13059-019-1630-6 (PMC6374913; doi:10.1186/s13059-019-1630-6)
Supplement: Supplementary file 3 — Protein alignments, corresponding Gblocks html file and CDS sequences for the lineage conserved gametophyte-biased genes. (ZIP 2109 kb) [file 13059_2019_1630_MOESM3_ESM.zip › Con_GA_alignments/Ec-01_011660.aa_ali.fasta-gb.htm]

Ec-01\_011660.aa.fasta.trans.aa\_ali.fasta


## Gblocks 0.91b Results

Processed file: **Ec-01\_011660.aa.fasta.trans.aa\_ali.fasta**  
Number of sequences: **2**  
Alignment assumed to be: **Protein**  
New number of positions: **492** (selected positions are underlined in blue)

```
                         10        20        30        40        50        60
                 =========+=========+=========+=========+=========+=========+
Ec-01_011660     MRCASYISPRQRDKSELCPDGQDPRQGGCSEAAERGTGAGDDLPRAPRRGGREGQGRAKR
MSTRG.46045.1    ------------------------------------------------------------
                                                                             


                         70        80        90       100       110       120
                 =========+=========+=========+=========+=========+=========+
Ec-01_011660     SFKVSGTLFELDAMYELIKPIGLGAYGVVVSALNTENGEKVAIKKITKAFDDLVDAKRIL
MSTRG.46045.1    --------------XQLIKPIGVGAYGVVISALNIENGQKVAIKKIARAFDDLVDAKRIL
                                 ############################################


                        130       140       150       160       170       180
                 =========+=========+=========+=========+=========+=========+
Ec-01_011660     REITLLRKFNHENIINIVDILVPPSPEEFEDVYIVSNLMETDLHRIINSKQELTPEHVQY
MSTRG.46045.1    REITLLRKFSHGNIISIVDILVPPSSEEFEDVYIVSELMETDLHRIINTRQELTPEHVQY
                 ############################################################


                        190       200       210       220       230       240
                 =========+=========+=========+=========+=========+=========+
Ec-01_011660     FIYQILRALKYMHSSNVLHRDLKPSNLLLNSNCELKVCDLGLARDIESGCQELTEYVVTR
MSTRG.46045.1    FLYQILRALKYMHSSNVLHRDLKPSNLLLNSNCDLKVCDLGLARDIESGCQELTEYVVTR
                 ############################################################


                        250       260       270       280       290       300
                 =========+=========+=========+=========+=========+=========+
Ec-01_011660     WYRAPEIMLGCHEYSKAIDVWSVGCIFAELMLRRPFFPGDNYIDQLTIICDKLGKPKEDE
MSTRG.46045.1    WYRAPEIMLACQEYSKAIDVWSVGCIFAELMLRRPFFPGDNYIDQLTIICDKLGKPKESE
                 ############################################################


                        310       320       330       340       350       360
                 =========+=========+=========+=========+=========+=========+
Ec-01_011660     LTFVSTEKARRFIMKLSATTPQRLRDQFPGTASDEALDLLAKMLEFSPETRISVEDALKH
MSTRG.46045.1    LTFVSTEKARRFILKLPSTTPKCLRDQFPSTASNEALDLLSKMLEFSPETRISVEDALRH
                 ############################################################


                        370       380       390       400       410       420
                 =========+=========+=========+=========+=========+=========+
Ec-01_011660     PFMQSYHTGNDEPSADFEFCFAFEEDVPTKHRLQELMWEEMRRYHPEQGEWKGKASPPTA
MSTRG.46045.1    PFMQNLHAVKEEPVANFEFCFAFESEVPSKHRLQELMWEEMRRYHPEQGPWRGKRSVP--
                 ##########################################################  


                        430       440       450       460       470       480
                 =========+=========+=========+=========+=========+=========+
Ec-01_011660     AAAKLIGSARNFGHLKRDTAADPLGRSPIKASPINSEGPNSSVGAVDIPPP---------
MSTRG.46045.1    ----ALSSPRAFGRLKGEATSTPLGRSPVKVGPAASGAGSSSVAAAAAPTDRGATPSVAT
                        ##########################################           


                        490       500       510       520       530       540
                 =========+=========+=========+=========+=========+=========+
Ec-01_011660     ---PGGGASASAERHGQPPAEGEPKTSRGLLSRAACSARPQTRRTSKRVAAAAAAAAAAA
MSTRG.46045.1    VDRPDGTAGGSPSIMPPPPERAHADESRSLLTHAVYSASEAPRRRTR--SMVVAEDPDAA
                    #########################################         #######


                        550       560       570       580       590       600
                 =========+=========+=========+=========+=========+=========+
Ec-01_011660     VQASVAPSNAAAVSEGRSSTPGGHRGLATVPAISTPTSAPYPPSSGSSSLSSSSASGDPL
MSTRG.46045.1    VGAPFG-INADTSVKGRRSTSTARPGIAMIPLDSTRTAAPC--SSSPVTMGTSSTAG---
                 ###     ################################   ##############   


                        610       620       630       640       650       660
                 =========+=========+=========+=========+=========+=========+
Ec-01_011660     GAAAAAPRARTRATARARVIKGRTTACPMAYSSSGSSCSENAGAQSVASATPDADSFLRR
MSTRG.46045.1    ------------------------------LTSSGS-----AG-----------------
                                                                             


                        670       680       690       700       710
                 =========+=========+=========+=========+=========+=====
Ec-01_011660     WAQTAAPVAGVKSPNPETVAANKRQRKGPGLETTRPANRPASPRARTCLHIARMP
MSTRG.46045.1    --------SGYLSPNAGSRAT----------------------------------
                          ###########
```

```
Parameters used
Minimum Number Of Sequences For A Conserved Position: 2
Minimum Number Of Sequences For A Flanking Position: 2
Maximum Number Of Contiguous Nonconserved Positions: 8
Minimum Length Of A Block: 10
Allowed Gap Positions: None
Use Similarity Matrices: Yes
```

```
Flank positions of the 7 selected block(s)
Flanks: [77  418]  [428  469]  [484  524]  [534  543]  [549  580]  [584  597]  [670  680]  

New number of positions in Ec-01_011660.aa.fasta.trans.aa_ali.fasta-gb:  492  (68% of the original 715 positions)
```
